# Research progress in lateral lymph node dissection for rectal cancer

**DOI:** 10.3389/fonc.2025.1631971

**Published:** 2025-09-30

**Authors:** Miao-Miao Dong, Zheng-peng Qian, Ji-Yong Lu, Jing-Jing Li, Yao-Chun lv, Shi-Yun Xu, Bin-Bin Du, De-Wang Wu

**Affiliations:** ^1^ Gansu Province Hospital Rehabilitation Center, Lanzhou, Gansu, China; ^2^ The First Clinical Medical College of Gansu University of Chinese Medicine, Lanzhou, Gansu, China; ^3^ Department of Anorectal Surgery, Gansu Provincial Hospital, Gansu Clinical Medical Research Center for Anorectal Diseases, Lanzhou, Gansu, China

**Keywords:** lateral lymph node dissection (LLND), low rectal cancer, total mesorectal excision (TME), neoadjuvant chemoradiotherapy (NCRT), robotic-assisted surgery

## Abstract

Lateral lymph node dissection (LLND), a critical surgical intervention for patients with low rectal cancer, plays a pivotal role in clinical practice. Its primary objective is to completely resect lateral pelvic lymph nodes, block the metastatic pathway of tumor cells, minimize the risk of postoperative recurrence, and provide a foundation for long-term survival. In Japan, LLND has become a standard surgical procedure for low rectal cancer and is widely applied. However, in Europe and North America, neoadjuvant chemoradiotherapy (nCRT) is regarded as the dominant treatment standard, which has led LLND to remain controversial and face doubts in clinical application. This article integrates the latest clinical research findings, explores the trajectory of technical evolution, analyzes clinical efficacy, objectively presents key points of academic controversy, and prospectively considers future developments. It aims to provide a comprehensive professional analysis for the rational application and continuous optimization of this technique in the treatment of low rectal cancer.

## 1 Historical background

LLND was first proposed and successfully performed by Japanese surgeons in the 1970s. Researchers in Japan observed that patients with low rectal cancer often presented with lateral pelvic lymph node metastasis, while total mesorectal excision (TME) alone could not completely remove these metastatic nodes, resulting in a persistently high local recurrence rate. An early Japanese clinical study enrolled 126 patients who underwent LLND, reporting a total lymph node involvement rate of 42% and a lateral lymph node involvement rate of approximately 9%. Among patients with low rectal cancer, 4 of 13 cases (31%) showed lateral lymph node involvement.

In 1977, Koyama reported that the 5-year survival rate of patients who underwent LLND was 45%, compared with 30% in patients who received conventional surgery. In 1982, Hojo and Koyama published the first English-language report on LLND, covering cases from 1962 to 1976. Since the introduction of LLND in 1969, the National Cancer Center Hospital (NCCH) in Japan has reported higher survival rates (71% vs. 59% for 5-year survival) (1). Based on these findings, Japanese surgeons proposed a combined surgical protocol of LLND with TME as the standard treatment strategy for rectal cancer (2).

In contrast to Japan, the understanding of lateral lymph node metastasis in rectal cancer has evolved continuously—from Miles’ initial description of lymphatic spread to subsequent studies exploring the role of LLND—resulting in repeated changes in surgical approaches and perspectives. In 1985, Glass et al. conducted a retrospective study of patients who underwent LLND (3). Based on vague indications (i.e., local extension or unfavorable histological grade), 75 patients underwent LLND and were compared with 2,266 patients who underwent conventional resection. No improvement in 5-year survival or local recurrence rates was observed in the LLND group, leading to the conclusion that patients did not benefit from the procedure. In 1992, Michelassi and Block reported on 73 patients who underwent conventional surgery and 64 patients who underwent extended pelvic lymphadenectomy; the local recurrence rate decreased from 16.4% to 9.4%, but the difference was not statistically significant (4). After this, only a few reports described the outcomes of Western patients undergoing LLND. Since the 1990s, rectal cancer treatment in Western countries has focused primarily on TME and (neo)adjuvant therapy, and this perspective gradually became the mainstream approach after the 1980s. However, whether laparoscopic LLND can provide additional benefits in specific high-risk populations has become a central point of academic debate (5, 6).

With years of continuous development and steady advances in diagnostic and therapeutic technology, China has also made significant progress in managing lateral lymph node metastasis in rectal cancer. From in-depth basic research to the accumulation of clinical experience, a series of new treatment theories and broad consensuses have gradually formed, leading to the establishment of a more comprehensive diagnostic and therapeutic system. This system emphasizes integrating precise diagnosis with personalized treatment strategies, which has greatly advanced treatment precision for patients with lateral lymph node metastasis and ensured that each patient receives an optimal plan tailored to their condition, thereby improving overall outcomes and prognosis (7).

### 1.1 Literature search and screening methods

To systematically summarize research progress in lateral lymph node dissection (LLND) for rectal cancer, we designed a search strategy following the principles of “comprehensiveness, standardization, and timeliness” to ensure the breadth and depth of included studies. Core search terms combined MeSH terms (Medical Subject Headings) and free text to cover the research object, intervention measures, and related technologies. The specific search term combination was: (“Rectal Neoplasms”[MeSH Terms] OR “Rectal Cancer”[Title/Abstract] OR “Lower Rectal Cancer”[Title/Abstract]) AND (“Lateral Lymph Node Dissection”[MeSH Terms] OR “LLND”[Title/Abstract] OR “Lateral Pelvic Lymph Node Dissection”[Title/Abstract]) AND (“Total Mesorectal Excision”[MeSH Terms] OR “TME”[Title/Abstract] OR “Neoadjuvant Chemoradiotherapy”[MeSH Terms] OR “nCRT”[Title/Abstract] OR “Laparoscopic Surgery”[Title/Abstract] OR “Robotic-Assisted Surgery”[Title/Abstract]). The searched databases included English databases such as PubMed, Cochrane Library, Web of Science Core Collection, and Embase (core publishing platforms for high-quality clinical studies, systematic reviews, and meta-analyses in rectal cancer surgery, ensuring completeness of Western research evidence) and Chinese databases such as China National Knowledge Infrastructure (CNKI), Wanfang Data Knowledge Service Platform, VIP Chinese Science and Technology Periodical Database (VIP), and China Biology Medicine Disc (CBM) (focusing on original studies and reviews by Chinese scholars related to LLND technological innovations [e.g., transanal-assisted surgery] and diagnosis and treatment consensuses [e.g., Chinese Expert Consensus on the Diagnosis and Treatment of Lateral Lymph Node Metastasis in Rectal Cancer (2024 Edition)]). The search timeframe was from the establishment of each database to May 2024, covering the 50-year development trajectory of LLND technology (from the first report in Japan in the 1970s to the latest multicenter studies in 2024) while ensuring inclusion of the latest clinical evidence (e.g., Dutch cohort studies and Chinese multicenter studies published in 2024).

The inclusion criteria were: original clinical studies related to LLND (randomized controlled trials, cohort studies, etc., with a sample size preferably ≥ 50 to ensure statistical power), systematic reviews, meta-analyses, and diagnosis and treatment consensuses; content focusing on LLND surgical techniques, imaging evaluation, treatment strategies, clinical efficacy, or complications; study populations consisting of patients with low rectal cancer (tumor inferior margin ≤ 5 cm from the anal verge) involving lateral lymph node evaluation or intervention; and literature in Chinese or English with complete abstracts or full texts. Exclusion criteria were: studies related to non–low rectal cancer (e.g., upper rectal cancer, colon cancer) or rectal surgery literature that did not explicitly mention “lateral lymph nodes”; animal experiments, basic mechanism studies (e.g., cellular-level mechanisms of lymph node metastasis), and technical short articles that only reported LLND procedures without clinical outcomes; duplicate publications (prioritizing the latest publication or the one with a larger sample size), conference abstracts (without complete data), and studies evaluated as “low quality” (e.g., randomized controlled trials without randomization methods, cohort studies with obvious selection bias).

Literature screening was conducted independently by two researchers. EndNote X9 software was used to manage retrieved citations exported from the database interface. First, records that clearly did not meet the inclusion criteria were excluded by reading the title and abstract, and records in dispute were marked “to be re-screened.” In the re-screening stage, full texts were obtained for the “to-be-re-screened” records and those passing the initial screening, and two researchers checked each article one by one against the inclusion/exclusion criteria. If disputes remained, a consensus was reached through discussion with a third researcher. For quality appraisal, different tools were used according to study type: the Cochrane Risk of Bias Assessment Tool was used for randomized controlled trials (e.g., JCOG0212), covering seven domains including random sequence generation, allocation concealment, and blinding; the Newcastle–Ottawa Scale (NOS) was used for cohort studies (e.g., Dutch national cohort), with scoring based on three domains—selection of study subjects, comparability between groups, and outcome measurement (a score ≥ 7 was considered high quality); and the AMSTAR 2 scale was used for systematic reviews/meta-analyses (e.g., meta-analyses related to LLND survival rate) to evaluate methodological quality. Only literature with moderate or higher quality was included for analysis to ensure the reliability of the review conclusions. For included studies, a standardized data extraction form recorded core information: first author and year of publication, study type, sample size, baseline characteristics (tumor stage, status of lateral lymph node metastasis), details of intervention measures (surgical procedure, nCRT regimen, imaging evaluation method), outcome indicators (local recurrence rate, 5-year overall survival [OS] and disease-free survival [DFS], complication rate), and key conclusions. After extraction, two researchers cross-checked the data to ensure no omissions or errors.

## 2 Technological advances

### 2.1 Academic analysis of traditional LLND and minimally invasive techniques

Traditional open LLND involves large incisions and extensive tissue manipulation, resulting in significant trauma. The long duration of such surgeries increases intraoperative blood loss and prolongs anesthesia exposure, further raising the risk of postoperative complications. Urinary and sexual dysfunction are common, particularly due to potential damage to the autonomic nerves controlling bladder and sexual function, which greatly affects postoperative quality of life (8). With advances in minimally invasive technology, the introduction of laparoscopic surgery and robotic-assisted surgery has reduced surgical trauma, decreased postoperative complications, and accelerated recovery. Laparoscopy, with its illumination and magnified imaging, has been widely adopted. Because the surgical field for rectal cancer is limited to the narrow pelvic cavity, laparoscopy offers certain advantages, with oncological outcomes comparable to those of open surgery (9, 10). Compared with the laparoscopic approach, emerging robotic-assisted surgery shows similar local recurrence rates and 5-year disease-free survival for robotic TME combined with LLND but achieves better long-term overall survival (11).

### 2.2 Advantages of laparoscopic and robotic-assisted surgery

Minimally invasive techniques, particularly laparoscopic and robotic-assisted surgery, have demonstrated advantages in reducing trauma, shortening surgical time, and accelerating recovery. Robotic-assisted surgery is especially effective in the anatomically complex pelvic region: 3D high-definition imaging and precise instrument control enable safer dissection, reducing nerve and vessel injury and significantly lowering postoperative urinary and sexual dysfunction (12, 13).

### 2.3 Comparative studies and outcomes

A large number of studies have shown that robotic-assisted surgery achieves comparable oncological outcomes to traditional open surgery in cancer treatment but has greater advantages in reducing complications and accelerating postoperative recovery (14–16). A study comparing robotic-assisted surgery and traditional open surgery showed that patients who underwent robotic surgery had faster postoperative recovery, shorter hospital stays, and better preservation of postoperative urinary and sexual function. These results indicate that robotic-assisted surgery has significant advantages in improving the postoperative quality of life of patients (17).

### 2.4 Transanal-assisted surgery (Transanal LLND)

Transanal-assisted LLND is an emerging surgical technique that accesses the pelvic region through the transanal route for lymph node dissection (18). The advantage of this technique lies in its ability to provide a better surgical field of view, especially in the pelvic region with complex anatomical structures. In 2018, the team led by Matsuda et al. (19) first explored transanal endoscopic lateral lymph node dissection, reported cases of combined transanal and transabdominal lateral lymph node dissection, and concluded that the combined transanal–transabdominal approach could significantly shorten the surgical time and reduce the surgical difficulty, particularly in the most distal part of the internal iliac artery. Chinese scholars conducted the first exploration and report of this surgery in 2019 (20). A recent study showed that the surgical time was shortened for robotic-assisted transabdominal LLND assisted by the transanal approach, and the incidence of postoperative urinary retention was low in both groups (21). Therefore, robotic-assisted abdominal LLND assisted by the transanal approach can be considered a promising treatment option for advanced low rectal cancer. Although this technique is still in the development stage, preliminary clinical studies have shown that it can reduce surgical trauma and improve surgical precision.

### 2.5 Summary

In summary, the transition from traditional open LLND to minimally invasive surgery—particularly robotic-assisted surgery—has brought about better surgical outcomes for patients. By reducing postoperative complications, accelerating recovery, and improving functional preservation, robotic-assisted surgery has demonstrated great potential in surgical procedures. Despite remaining challenges, with the advancement of technology and optimization of medical resources, robotic-assisted surgery will play a more extensive role in the future.

## 3 Application value of imaging prediction in preoperative evaluation and postoperative recurrence of lateral lymph nodes in rectal cancer

Preoperative imaging evaluation is an important basis for deciding whether to perform LLND. Early imaging diagnosis did not bring specific benefits to patients with lateral lymph node metastasis; however, with the advancement of imaging technologies such as magnetic resonance imaging (MRI) and positron emission tomography–computed tomography (PET–CT), surgeons can more accurately evaluate the presence of lymph node metastasis. Through detailed analysis of imaging results, surgeons can better determine which patients need LLND, thereby avoiding unnecessary surgeries and reducing surgical risks.

Imaging methods are not only used for the initial evaluation of lateral lymph node metastasis in patients. Malakorn et al. retrospectively analyzed the response of lateral lymph nodes (LLN) on restaging MRI after chemoradiotherapy (CRT) in 64 patients with suspected LLN metastasis who underwent LLND at a single center from 2006 to 2017 (22). This clinical study used imaging methods to reclassify LLN after treatment, providing insights for clinical treatment strategies. A study from Eastern countries showed that in MRI restaging, LLN with a short axis < 5 mm had no pathological positivity, while the postoperative pathological positive rate of LLN with a short axis > 5 mm could reach 64.7% (23). This study indicated that LLND may only be beneficial for patients with LLN > 5 mm, and defined the cutoff value for changes in LLN size after CRT as 5 mm, which provides a reference for surgeons in deciding whether to perform LLND.

A Dutch cohort study on predicting patient prognosis used imaging methods to analyze the number and imaging features of lateral lymph nodes in patients with locally advanced (cT3–T4) rectal cancer. The malignant features of lateral lymph nodes were divided into four aspects: number of lateral lymph nodes, presence of malignant features (internal heterogeneity, irregular margins, disappearance of fat hilum, and round shape), disappearance on follow-up MRI scans, and short-axis size. The results showed that the 4-year lateral local recurrence (LLR) rate was 0% in patients with a single malignant feature, while the recurrence rate was 17% in patients with multiple malignant features; among patients with multiple malignant features and small (< 5 mm) lateral lymph nodes, the 4-year local recurrence rate was 20% if the malignant features persisted; the 4-year local recurrence rate was 8% in patients with medium-sized LLN (5.0–6.9 mm) with at least 1 malignant feature; the 4-year LLR rate was 28% in patients with multiple enlarged LLN, compared with 11% in patients with a single enlarged LLN (24). This latest report provides new ideas and directions for the stratified management of lateral lymph node metastasis and offers clinical value for deciding whether to perform LLND during the follow-up of patients with locally advanced rectal cancer.

## 4 Changes in treatment modalities for lateral lymph node metastasis

In the past, there have been many debates and divergent views on the treatment of lateral lymph node metastasis in rectal cancer. Early Western perspectives held that lateral lymph node dissection caused significant damage to patients’ urinary and sexual function, was technically difficult to perform, and showed no significant survival benefit (25). A study involving 1,216 patients from the Lateral Lymph Node Consortium showed that patients with LLN (short axis ≥ 7 mm) detected at the initial examination had a 19.5% risk of LLR, and neoadjuvant therapy combined with TME failed to provide significant benefits (26). Therefore, Western countries shifted their focus on lateral lymph node metastasis to the neoadjuvant therapy stage (nCRT) and achieved certain clinical effects. In contrast, early Eastern countries—primarily based on Japanese research—recommended the TME + LLND approach for treating low rectal cancer and did not advocate radiotherapy. Japan’s advocacy of prophylactic LLND derives from early research showing that the probability of lateral lymph node metastasis increases with advancing tumor stage (27). Thus, Japanese scholars recommend prophylactic LLND and established TME + LLND as the standard treatment.

With in-depth research on locally advanced rectal cancer and improved diagnostic and therapeutic capabilities in both Eastern and Western countries, certain conclusions have been drawn. Western studies have shown that nCRT alone cannot completely eliminate lateral lymph node metastasis; in patients with suspected preoperative metastasis, the recurrence rate after treatment can reach 80% (23). This suggests that for this subset of patients, nCRT combined with TME alone may be an ineffective strategy (28, 29). However, recent results have shown that combining TME + LLND with nCRT can more effectively prevent local recurrence. Further research has demonstrated that nCRT + TME + LLND improves DFS and OS in patients with locally advanced low rectal cancer compared with TME + LLND alone. For patients initially diagnosed with rectal cancer and suspected LLNM, the modality of nCRT combined with TME + LLND may be a viable option (30).

## 5 Clinical efficacy

### 5.1 Reduction in local recurrence rate

A study by Zhou Sicheng et al. showed that pathological lateral lymph node metastasis (LLNM) is an independent risk factor for decreased overall survival (OS) and disease-free survival (DFS) in rectal cancer patients (31). A Japanese study found that the most common site of local recurrence in patients with locally advanced low rectal cancer is the lateral pelvis, with approximately 50% experiencing recurrence in this region; such metastasis affects both treatment outcomes and survival (32). Clinical studies have demonstrated that LLND can significantly reduce local recurrence, and for patients with lateral lymph node metastasis, LLND combined with TME is considered the most effective surgical protocol (33). Therefore, after years of development and debate in Eastern and Western countries, the latest research data suggest that for patients with rectal cancer and lateral lymph node metastasis, LLND can provide clinical benefits.

### 5.2 Impact on survival rate

In a randomized controlled study (JCOG0212), 701 patients with stage II–III low rectal cancer were randomly divided into the TME group and the TME + LLND group. The results showed no statistically significant differences in overall survival (OS) and 5-year disease-free survival (DFS) between the two groups (34). Although LLND showed a significant advantage in terms of local recurrence rate, its impact on long-term OS and DFS remains unclear.

A recent meta-analysis supplemented data on long-term survival after LLND (11). The analysis showed that OS in the LLND group was not significantly prolonged, but the 5-year survival rate was higher after LLND. However, the LLND group also had longer surgical duration and more severe urinary dysfunction, suggesting that LLND has limited effect on improving long-term survival. Other studies have noted that LLND may prolong survival in certain high-risk patients; specifically, for those with ineffective neoadjuvant therapy or extensive lymph node metastasis, LLND may provide additional survival benefits (11).

Akiyoshi et al. studied the clinical efficacy of LLND after CRT. This trial enrolled 127 patients with stage II–III rectal cancer below the peritoneal reflection who underwent LLND, of whom 38 received CRT + TME + LLND and 89 received only CRT + TME. Pathological metastasis was confirmed in 25 of 38 patients (65.8%) after LLND (23). There was no difference in recurrence rate between the two groups (2.7% in the LLND group vs. 7.1% in the TME group; p = 0.27). Therefore, even if patients with suspected LLND have developed lateral lymph node metastasis, the LLR rate of these patients is similar to that of the non-LLN group when LLND is performed.

In Australia and the United States, patients with an LLN short axis of 5 mm from the MD Anderson Cancer Center in Houston, Texas, who received CRT + TME + LLND were compared with patients from other Western centers who received only CRT + TME. The results showed that the LLR rate was 3% in the LLND group and 11% in the non-LLND group, indicating that Western patients who undergo LLND in addition to CRT and TME may also achieve improved oncological outcomes (35).

(**Table 1**: Comparison of survival outcomes in different treatment strategies for lateral lymph node metastasis in rectal cancer).

**Table 1:** Comparison of survival outcomes in different treatment strategies for lateral lymph node metastasis in rectal cancer.

Study	Participants	Intervention groups	Main findings
JCOG0212 (37)	701 patients with stage II - III low rectal cancer	TME group and TME + LLND group	LLND had a significant advantage in local recurrence rate, but its impact on long-term survival (OS) and disease - free survival (DFS) was unclear. The LLND group had longer operative time and more severe urinary dysfunction, with limited impact on improving long - term survival.
Meta - analysis study (11)	N/A (data - supplementing study)	N/A (compared LLND in general)	- Overall survival in the LLND group was not significantly prolonged, but the 5 - year survival rate was higher after LLND.
Akiyoshi et al. study (23)	127 patients with stage II - III rectal cancer below the peritoneal reflection	38 patients receiving CRT + TME + LLND treatment and 89 patients receiving CRT + TME only	No difference in recurrence rates between the two groups (2.7% in the LLND group and 7.1% in the TME group; p < 0.27).
Comparison between MD Anderson Cancer Center and other Western cancer centers (35)	Patients with LLN short axis ≥ 5mm from MD Anderson Cancer Center (treated with CRT + TME + LLND) and patients from other Western cancer centers (treated with CRT + TME only)	CRT + TME + LLND group and CRT + TME only group	The LLR rate was 3% in the LLND group and 11% in the non - LLND group, suggesting that Western patients undergoing LLND clearance in addition to CRT and TME may achieve some effectiveness in oncologic outcomes.

## 6 Controversies and challenges

### 6.1 Complication risks

One of the main controversies regarding LLND is the high risk of surgery-related complications. The pelvic anatomy is complex, and LLND is prone to damaging surrounding nerves and blood vessels, leading to postoperative urinary and sexual dysfunction. Studies have shown that LLND is associated with longer surgical duration, greater intraoperative blood loss, and a relatively higher incidence of postoperative complications.

After TME alone, the incidence of erectile dysfunction was 68% in the TME group and 79% in the TME + LLND group (p = 0.15) (34, 36); the incidence of early urinary dysfunction was 58% in the TME group and 59% in the TME + LLND group. This indicates that TME alone also carries a high risk of sexual and urinary dysfunction, and LLND does not appear to increase this risk (37). A meta-analysis including 18 studies showed that compared with TME alone, TME + LLND resulted in worse functional outcomes and a higher risk of postoperative complications: the odds ratio (OR) for urinary dysfunction was 6.66 (p < 0.001), and the OR for sexual dysfunction was 9.67 (p < 0.002). However, in some studies, autonomic nerves were resected, and subgroup analysis of nerve-sparing techniques was not possible. Therefore, the potential risks of LLND in certain patients need to be weighed against its clinical benefits. In summary, the essence of the controversy over whether to perform LLND lies in the purpose of the surgery—i.e., whether the surgery is for prophylactic lateral lymph node dissection or for dissection once substantial lateral lymph node metastasis is detected.

Second, whether patients can achieve improved long-term survival after LLND still requires large-scale, multicenter clinical studies. Current evidence only indicates that patients with ineffective neoadjuvant therapy and extensive lymph node metastasis may obtain survival benefits, but there is no clear proof that LLND improves long-term survival overall.

Third, the alternative role of neoadjuvant therapy: in Western countries, nCRT combined with TME is considered an effective method for managing lateral lymph node metastasis. Many Western studies have suggested that nCRT achieves comparable efficacy to LLND and may even eliminate the need for additional dissection in some cases. Therefore, whether LLND should be routinely applied remains an important focus of academic debate (8, 13).

### 6.2 Impact of neoadjuvant therapy

In Western countries, the regimen of nCRT combined with TME is widely accepted and considered an effective alternative to LLND. Some studies have reported that neoadjuvant therapy can significantly reduce the local recurrence rate and lessen the need for LLND. However, for patients with poor response to neoadjuvant therapy, LLND may still play an important role, particularly when imaging shows the presence of lateral lymph node metastasis (37).

A Chinese study reported that the treatment modality of neoadjuvant therapy combined with TME + LLND had a median follow-up period of 37 months, and the 3-year DFS rate of the entire cohort was 74.8%. This study did not confirm clinical benefits of combining LLND after nCRT in patients with locally advanced rectal cancer (LARC) and clinically suspected lateral pelvic lymph node metastasis (LPNM) (38). The conclusions of a recent multicenter study were similar: the results showed that LLND without nCRT is effective and sufficient in preventing local recurrence in patients with LPN metastasis (39). Therefore, while nCRT may increase local disease control in patients with LARC and lateral lymph node metastasis, there is no evidence that it prolongs long-term survival.

Combination therapy strategies: Some trials, such as the Janus Rectal Cancer Trial, have investigated the efficacy of TME with or without LLND after long-course chemoradiotherapy (40). These studies aim to evaluate the efficacy of combining LLND with neoadjuvant therapy and optimize treatment regimens; however, long-term survival data have not yet been reported.

## 7 Future directions

### 7.1 Personalized treatment strategies

With the development of precision medicine, the application of LLND is gradually moving toward personalization. By integrating imaging evaluation, tumor staging, and the patient’s overall condition, surgeons can better identify patients most suitable for LLND. This personalized strategy can reduce the risk of unnecessary surgery and provide optimal treatment for high-risk patients.

### 7.2 Multidisciplinary collaboration

Future LLND treatment strategies will rely more on multidisciplinary collaboration. Through the joint efforts of surgeons, oncologists, radiation therapists, and imaging specialists, more comprehensive treatment plans can be provided. This collaboration can improve surgical success rates and assist in postoperative management of complications and adjuvant therapy.

## 8 Conclusion

As a treatment modality for low rectal cancer, LLND has significant therapeutic effects in high-risk patients with lateral lymph node metastasis. However, its complexity and the risk of postoperative complications have led to ongoing controversies in clinical practice. The priority-approach surgery developed in China has improved the safety of LLND and reduced the incidence of postoperative complications.

Neoadjuvant chemoradiotherapy (nCRT) combined with TME is an effective method for managing lateral lymph node metastasis, but evidence is lacking on whether it improves long-term survival. With the continuous development of minimally invasive and imaging technologies, LLND will likely become more precise and personalized. Although current studies support the advantages of robotic-assisted surgery over traditional LLND, further exploration is required. For example, long-term follow-up studies on functional recovery (urinary and sexual function) are needed. In addition, the high cost of robotic surgery limits its use in resource-constrained areas, making cost-effectiveness analyses essential.

To further optimize outcomes, surgeon training should be strengthened—particularly the use of simulation and virtual reality (VR), which can help surgeons master robotic techniques more quickly and improve efficiency. Future research should also refine the indications for LLND and promote multidisciplinary collaboration to provide better treatment plans for patients.
